# Supplementary material for: Consumption of animal source food and associated factors among pregnant women in eastern Ethiopia: A community-based study
Source: PLoS One. 2022 Jun 17;17(6):e0270250. doi: 10.1371/journal.pone.0270250 (PMC9205500; doi:10.1371/journal.pone.0270250)
Supplement: Attachment — Submitted filename: Reviewed article PONE-D-21-17077.pdf [file pone.0270250.s001.pdf]

PLOS ONE

Dietary Practices and Associated Factors Among Pregnant Women in Haramaya District, Eastern Ethiopia

--Manuscript Draft--

**Manuscript Number:**	PONE-D-21-17077
**Article Type:**	Research Article
**Full Title:**	Dietary Practices and Associated Factors Among Pregnant Women in Haramaya District, Eastern Ethiopia
**Short Title:**	Dietary Practices and Associated Factors Among Pregnant Women
**Corresponding Author:**	Meseret Belete Fite, PhD candidate<br>Wollega University<br>Nekemte, Oromia ETHIOPIA
**Keywords:**	Prevalence; Dietary Practice; Pregnant Women; Haramya; Ethiopia
**Abstract:**	Introduction<br><br>Dietary practice in pregnancy has continued to get global attention as pregnancy is considered a critical period in the life-cycle owed to increase the metabolic and physiological demand. However information on dietary practice among pregnant women in Ethiopia, particularly of those resident of rural are a is limited. Therefore,this study aimed to assess dietary practice and its determinants among pregnant women in Harmaya district<br><br>Methods<br><br>Sectional study design was conducted involving 448 pregnant women from January 5 to February 12, 2021. Data was collected with interviewers-administered questionnaires by well-trained health professionals. Validated Food frequency questionnaires was used to asses dietary practice. Four measures including; dietary diversity, food variety, consumption of animal source foods and frequency of meal were used to measure the dietary practices of pregnant women. A Poisson regression model with robust variance estimation was used to investigate the independent variable's association with dietary practice. An adjusted prevalence ratio with 95% confidence interval was reported to show associations.<br><br>Results<br><br>The prevalence of dietary practice among pregnant women was 15.18% (95% CI=11.96-18.84) . From total respondents 29.46%, 37.5% and 24.7% of them had high dietary diversity, high food variety score and high consumption of animal source food respectively. Dietary practice was more prevalent among merchant women (APR=2.07; 95% CI 1.071- 4.016 and those whose husband have high school and above educational level (APR=1.96; 95% CI=1.1.063-3.460) .However, the prevalence of dietary practice was significantly lower among those who reported chat chewing (APR=0.58; 95% CI= 0.370- 0.901) and avoiding food intake (APR= 0 .36; 95% CI=0.200- 0.651) .<br><br>Conclusion<br><br>The the present study showed that dietary practice of pregnant women is sub-optimal. Lower prevalence was observed on women who reported chat chewing and restricted dietary consumption during pregnancy. Nutrition policy, programs and interventions should aimed at encouraging prenatal dietary guidance and counselling are recommended .
**Order of Authors:**	Meseret Belete Fite, PhD candidate
	Kedir Teji Roba
	Lemesa Oljirra Lemesa

Powered by Editorial Manager® and ProduXion Manager® from Aries Systems Corporation

	Abera Kenay Tura<br>Tesfaye Assebe Yadeta
**Additional Information:**	
**Question**	**Response**
**Financial Disclosure**<br><br>Enter a financial disclosure statement that describes the sources of funding for the work included in this submission. Review the submission guidelines for detailed requirements. View published research articles from *PLOS ONE* for specific examples.<br><br>This statement is required for submission and **will appear in the published article** if the submission is accepted. Please make sure it is accurate.<br><br>**Unfunded studies**<br>Enter: *The author(s) received no specific funding for this work.*<br><br>**Funded studies**<br>Enter a statement with the following details:<br>• Initials of the authors who received each award<br>• Grant numbers awarded to each author<br>• The full name of each funder<br>• URL of each funder website<br>• Did the sponsors or funders play any role in the study design, data collection and analysis, decision to publish, or preparation of the manuscript?<br>• **NO** - Include this sentence at the end of your statement: *The funders had no role in study design, data collection and analysis, decision to publish, or preparation of the manuscript.*<br>• **YES** - Specify the role(s) played.<br><br>* typeset	This study was fully funded by Haramaya University, Ethiopia. The funder has no role in conception, design of the study, statistical analysis, result interpretation and in writing up the manuscript. The funding institution has no role in the publication consent or approval.
**Competing Interests**<br><br>Use the instructions below to enter a competing interest statement for this submission. On behalf of all authors, disclose any competing interests that could be perceived to bias this work—acknowledging all financial support and any other relevant financial or non-	The authors have declared that no competing interests exist.

financial competing interests.<br><br>This statement is required for submission and **will appear in the published article** if the submission is accepted. Please make sure it is accurate and that any funding sources listed in your Funding Information later in the submission form are also declared in your Financial Disclosure statement.<br><br>View published research articles from *PLOS ONE* for specific examples.<br><br>**NO authors have competing interests**<br><br>Enter: *The authors have declared that no competing interests exist.*<br><br>**Authors with competing interests**<br><br>Enter competing interest details beginning with this statement:<br><br>*I have read the journal's policy and the authors of this manuscript have the following competing interests: [insert competing interests here]*<br><br>* typeset	
**Ethics Statement**<br><br>Enter an ethics statement for this submission. This statement is required if the study involved:<br><br>• Human participants<br>• Human specimens or tissue<br>• Vertebrate animals or cephalopods<br>• Vertebrate embryos or tissues<br>• Field research<br><br>Write "N/A" if the submission does not require an ethics statement.<br><br>General guidance is provided below. Consult the submission guidelines for	This study was conducted in agreement with the Declaration of Helsinki-Ethical principle for medical research involving the human subjects. Accordingly, ethical approval letter was obtained from Haramaya University Institutional Research Ethics and Review Committee (IRERC) with a reference number of (IHRERC/266/2020) before commencement of data collection

detailed instructions. **Make sure that all information entered here is included in the Methods section of the manuscript.**<br><br>**Format for specific study types**<br><br>**Human Subject Research (involving human participants and/or tissue)**<br>• Give the name of the institutional review board or ethics committee that approved the study<br>• Include the approval number and/or a statement indicating approval of this research<br>• Indicate the form of consent obtained (written/oral) or the reason that consent was not obtained (e.g. the data were analyzed anonymously)<br><br>**Animal Research (involving vertebrate animals, embryos or tissues)**<br>• Provide the name of the Institutional Animal Care and Use Committee (IACUC) or other relevant ethics board that reviewed the study protocol, and indicate whether they approved this research or granted a formal waiver of ethical approval<br>• Include an approval number if one was obtained<br>• If the study involved *non-human primates*, add *additional details* about animal welfare and steps taken to ameliorate suffering<br>• If anesthesia, euthanasia, or any kind of animal sacrifice is part of the study, include briefly which substances and/or methods were applied<br><br>**Field Research**<br><br>Include the following details if this study involves the collection of plant, animal, or other materials from a natural setting:<br>• Field permit number<br>• Name of the institution or relevant body that granted permission	
**Data Availability**<br><br>Authors are required to make all data underlying the findings described fully available, without restriction, and from the time of publication. PLOS allows rare	Yes - all data are fully available without restriction

exceptions to address legal and ethical concerns. See the PLOS Data Policy and FAQ for detailed information.<br><br>A Data Availability Statement describing where the data can be found is required at submission. Your answers to this question constitute the Data Availability Statement and **will be published in the article**, if accepted.<br><br>**Important:** Stating 'data available on request from the author' is not sufficient. If your data are only available upon request, select 'No' for the first question and explain your exceptional situation in the text box.<br><br>Do the authors confirm that all data underlying the findings described in their manuscript are fully available without restriction?	
**Describe where the data may be found in full sentences. If you are copying our sample text, replace any instances of XXX with the appropriate details.**<br><br>• If the data are **held or will be held in a public repository**, include URLs, accession numbers or DOIs. If this information will only be available after acceptance, indicate this by ticking the box below. For example: *All XXX files are available from the XXX database (accession number(s) XXX, XXX.*).<br>• If the data are all contained **within the manuscript and/or Supporting Information files**, enter the following: *All relevant data are within the manuscript and its Supporting Information files.*<br>• If neither of these applies but you are able to provide **details of access elsewhere**, with or without limitations, please do so. For example:<br><br>*Data cannot be shared publicly because of [XXX]. Data are available from the XXX Institutional Data Access / Ethics Committee (contact via XXX) for researchers who meet the criteria for*	All relevant data are within the manuscript

access to confidential data.<br><br>*The data underlying the results presented in the study are available from (include the name of the third party and contact information or URL).*<br>• This text is appropriate if the data are owned by a third party and authors do not have permission to share the data.<br><br>* typeset	
Additional data availability information:	

Powered by Editorial Manager® and ProduXion Manager® from Aries Systems Corporation

Click here to access/download;Manuscript;Manuscript of dietary Practice.docx

Dietary Practices and Associated Factors Among Pregnant Women in Haramaya District, Eastern Ethiopia

Meseret Belete Fite[1*,] Kedir Teji Roba[2&] Lemessa Oljira[2&.] Abera Kenay Tura[2&.] Tesfaye Assebe Yadeta[2&.]
[1]Department of Public Health, Institute of Health Sciences, Wollega University, Nekemte, Ethiopia
[2]Department of Public Health, School of Public Health, College of Health and Medical Sciences, Haramaya University, Harar, Ethiopia
* **Corresponding author**
E-mail: meseretphd2014@gmail.com
And Those authors listed above are also contributed equally to this work.

Abstract

Introduction: Dietary practice in pregnancy has continued to get global attention as pregnancy is considered a critical period in the life-cycle owed to increase the metabolic and physiological demand. However information on dietary practice among pregnant women in Ethiopia, particularly of those resident of rural are a is limited. Therefore,this study aimed to assess dietary practice and its determinants among pregnant women in Harmaya district

Methods: Sectional study design was conducted involving 448 pregnant women from January 5 to February 12, 2021. Data was collected with interviewers-administered questionnaires by well-trained health professionals. Validated Food frequency questionnaires was used to asses dietary practice. Four measures including; dietary diversity, food variety, consumption of animal source foods and frequency of meal were used to measure the dietary practices of pregnant women. A Poisson regression model with robust variance estimation was used to investigate the independent variable's association with dietary practice. An adjusted prevalence ratio with 95% confidence interval was reported to show associations.

Results: The prevalence of dietary practice among pregnant women was 15.18% (95% CI=11.96-18.84) . From total respondents 29.46%, 37.5% and 24.7% of them had high dietary diversity, high food variety score and high consumption of animal source food respectively. Dietary practice was more prevalent among merchant women (APR=2.07; 95% CI 1.071- 4.016 and those whose husband have high school and above educational level (APR=1.96; 95% CI=1.1.063-3.460) .However, the prevalence of dietary practice was significantly lower among those who reported chat chewing (APR=0.58; 95% CI= 0.370- 0.901) and avoiding food intake (APR= 0 .36; 95% CI=0.200- 0.651) .

Conclusion: The the present study showed that dietary practice of pregnant women is sub-optimal. Lower prevalence was observed on women who reported chat chewing and restricted dietary consumption during pregnancy. Nutrition policy, programs and interventions should aimed at encouraging prenatal dietary guidance and counselling are recommended .

Keywords: Prevalence, Dietary Practice, Pregnant Women, Haramya, Ethiopia

Introduction

Healthy and balanced diet is essential for all human beings for the appropriate working of the body system (1) Pregnancy is a critical period in the life-cycle owed to increase the metabolic and physiological demands and; adequate nutrition is essential to meet these conditions . At conception many women lack sufficient micronutrients stores to meet increased requirement of pregnancy (2 , 3). Inappropriate dietary practice in pregnancy is one of the factors that contribute to under nutrition and micronutrients deficiency which may also result to poor birth outcome (4). In unindustrialized world, inappropriate dietary practice fallouts as of inadequate access to food sources or after poor knowledge of the essence of virtuous quality and amount diet (5-8). Imbalanced macronutrients, inadequate micronutrient intakes and predominantly plant-based diets were common features of the diet of pregnant women in developing countries (9).

In low-middle income countries good dietary diversity practice of pregnant women vary within a country ranging from 20% in the Pakistan to 70% in the India (10). Most of pregnant women in Sub-Saharan Africa have insufficient dietary consumption and do not meet their Recommended Dietary Allowances (RDA) (11). Evidences suggested that appropriate dietary practice of pregnant women in Ethiopia diverge extending from 19.9% in the Gojjam (5) and 40.1% in the Gonder (6). Most of the researches used meal frequency to evaluate the dietary practice of pregnant women, whereas some used tools on nutrient content of the foods. In developing country context where the pregnant women commonly did not identify the nutrient content food, the question on nutrient content food is not frequently used to assess dietary intake. In low-income county, where people habitually served diets from communal crossbow; dietary diversity, food Variety score, consumption of animal source score and meal frequency are measures usually applied to gauge dietary practice of pregnant women (12-17)

Women's biological, social, physiological, economic, environmental and psychological characteristics are a complex set of determinants that affect their dietary practice in pregnancy. From these determinants, except the biological others can be modified (18). From the review of different literature, the socio-economic determinant of dietary practice of pregnant women is well covered by former investigators. However, environmental and psychological factors which influences dietary practice during pregnancy are poorly documented in Ethiopian context and the problem is undetected particularly

in the eastern part of the county. Moreover, the current prevalence of dietary practice and it's the socio-economic, environmental and psychological factors in pregnancy is not well-known in this setup empirically. Therefore, the problem could be undetectable to policy makers.

In the present study, we aimed to comprehensively assess dietary practice and its determinants among pregnant women. First, we examined socio-economic and obstetric characteristics of pregnant women. Then, we identified predictors of dietary practice among pregnant women.

Methods

Description of study area

The present study was conducted in Haramaya district, Eastern Ethiopia. It is 500 KM away from the capital city, Addis Ababa. The district is also, divided into 33 rural and 2 urban "kebeles" which are the lowermost unit of administrative in Ethiopia. Haramaya district is one of a few Health Demographic Surveillance and Health Research (HDS-HRC) field sites established in the country. Among all kebles of the district, twelve (12) rural kebeles are HDS-HRC field site. The altitude of the district ranges from 1400 to 2340 meters above sea level. The district has a total population of 271,018, of which 51% were females. According to 2007 national census of Ethiopia, the number of projected pregnant women was 5252. The livelihood of the people in the district is built on mixed farming by major crop production. Chat is the dominant crop broadly produced in the area. Green cabbage, kale, tomato, cannonball cabbage and peanut are foodstuff commonly produced in the area. Goat and sheep are the are common livestock in the district. (19)

Study design and period

Community based cross sectional study design was conducted in January 5 to February 12, 2021.

Source population and study population.

All pregnant women who were resided for at least six months in the district during study period were source population of this study. Whereas, pregnant women who were randomly selected from selected kebeles and included in this study were the study population of the current study.

Inclusion and exclusion criteria

Participants were a parts a of pregnancy surveillance initiated in HDS-HRC. For the reason that dietary practice is affected by the local social and cultural value, all pregnant women who were lived minimum of six months in the district were involved in this study. However, all pregnant women with reported acute and chronic illnesses, seriously ill and unable to communicate during study period were excluded.

Sample size determination and sampling procedures

The sample size required and adequate for estimating the determinants of dietary practices of pregnant women was computed using single and double population proportion formula with their corresponding assumption. Nevertheless, sample size computed by double population proportion formula using the following assumption gave the largest sample size in this study: 95% Confidence Interval level, power 80%, Ratio of 1:1 between exposed and unexposed subjects, 19% prevalence of third trimester (Unexposed) stage of pregnancy, 8.9% of first trimester (Exposed) stage of pregnancy (20) 5% marginal error and 10% non-response rate. The final sample size calculated was 450. Haramaya district was purposefully selected as study area. Out of total kebeles of the district 12 of them are HDS-HRC field site. Conventionally, these kebeles were selected as HDS-HR field site based on probability proportional to size (PPS) sampling technique. Moreover, simple random sampling method was applied to select eight (8) kebeles from HDS-HRC field site kebeles. The determined sample size was proportionally allocated to the selected kebeles. Since the participants were the parts of pregnancy surveillance of HDS-HRC, list of all pregnant women was obtained from HDS-HRC office and, then we used as sampling frame. Based on the proportional to size allocation of their respective kebeles, all eligible pregnant women were selected with simple random sampling methods/lottery methods by using their list from HDS-HRC registration. House to house survey was implemented to identify eligible pregnant women in selected kebeles. Finally, all eligible women were included in the study.

Data collection and Measurement

Data was collected with interviewers-administered questionnaires by well-trained health professionals. Structured questionnaires were employed to collect data on socio-economic, obstetric, maternal perception, food consumption, dietary knowledge, attitude and practices of pregnant women. These questionnaires were fist prepared in English language and then translated to local language (Afan Oromo). After translation, it's the consistency was checked by another person

who speaks both languages. We pre-tested the questionnaires on 10% of the sampled pregnant women of the district, that were not included in the main study and modification was done based on the pre-test observations.

Four measures including; dietary diversity, food variety, consumption of animal source foods and frequency of meal were used to measure the dietary practices of pregnant women (21-23). We used formerly validated Food Frequency Questionnaires (FFQ) which contained 27 commonly consumed food items consumed by the community of the district to asses dietary diversity (23-25). Initially, the list of food items was established grounded on wide-ranging consultation of key informants who are from the study area and who knew the culture and local language on types and food groups usually consumed. We pre-tested the Food frequency questionnaires on 10% of the sampled pregnant women of the district, that were not included in the main study and modification was done based the pre-test observations. For the FFQ, the women were invited to tale about the consumption of each food per day, per week or per month using the past three months in consideration of the difference of dietary consumption within a day of a week into account (26-27)

Considered the greater difference of dietary practice in the local community over the day of the week, the intake of each food item per a day (6 , 23) was not taken as a cut-off point to label consumers. Nevertheless, pregnant women were defined as a "consumer" of food items if they had consumed food items minimum once over a period of a week (21 , 22) Food items of food frequency questionnaires was grouped into ten food groups: cereal, white roots and tubers, pulse and legumes, nuts and seeds, dark green leafy vegetables, other vit A-rich fruits and vegetables, meat, fish and poultry, dairy and dairy product, egg, other vegetables and other fruits (26). The sum of each of food groups the pregnant women consumed over period of week were calculated to analysis dietary diversity score (DDS). Furthermore, we converted dietary diversity score into terciles and the highest tercile was used to label "high" dietary diversity score, whereas the both lower terciles combined were defined as" low" dietary diversity score. Food variety score (FVS) is the frequency of individual food items consumed in the reference period. Therefore, it was estimated by the intake of each the individual 40 food items over seven days (27) , with maximum of FVS fourth. Then mean FVS was calculated ad pregnant women with FVS greater than means were labeled as having "high" food variety score, whereas, those with FVS lower than the means were defined as having "low" FVS. Consumption of animal source foods (ASF) was estimated by counting the frequency of each animal source foods the pregnant women ate over a reference period, which was seven day. We converted animal source foods score into terciles and the highest tercile was used to label "high's, while the two lower terciles combined were defined as" low" ASF.

Data Quality Assurance

Two days rigorous and extensive training with the final version of the questionnaires was given for data collectors prior to pre-test. pre-tested the questionnaires on 10% of the sampled pregnant women of the district, that were not included in the main study and modification was done based on the pre-test observations. The supervisors kept the alleyway of the filed procedures and checked the completed questionnaires daily to approve the accuracy of data collected and the research team managed an overall work of data collection

Analysis

Data were cleaned, coded and entered in double by using EPiData version 3.1 software, checked for missing and outliers and analyzed using STATA version 14 (College Station, Texas 77845 USA) statistical software. The outcome variable was dichotomized as dietary Practice=1 (Appropriate) and dietary Practice=0 (Inappropriate). To assess the predictors of DDS and ASF bivariate analyses were conducted. Multivariable analyses were carried out to identify determinants of dietary practice among pregnant women. Thus, Poisson regression analysis model with robust variance estimate was fitted to identify predictors of dietary practice of women. For multivariable analyses, only variables that displayed a $p< 0.2$ in the bivariate analyses were entered in the adjusted model. The backward regression was fitted with selected socio-economic and fertility related variables. The results are presented as adjusted prevalence ratios (APRs) with 95% CI. The statistical level of significance was set at alpha=5%. The explanatory variables were examined for multi-collinearity before taking them into multivariable models using correlation matrix for the regression coefficients, using the standard errors, and variance inflation factors value. Possible interactions between covariates were tested. Akaike's information criterion (AIC) and Bayesian information criterion (BIC) were used to test for model fitness.

To estimate the economic level of the families, wealth index was employed. The wealth dispersion was generated by applying principal component analysis. The index was calculated based on the ownership of latrine, ownership agricultural land and size, selected household asset, quantity of livestock and source of water used for drinking, that was to 41 household variables (). Then, we identified five principal components having eigenvalues greater than one. The wealth index quintile was co[illegible]ted from wealth score of the households. Finally, the computed quintile was used to label the households wealth status to poorest, poor, middle, rich and richest category. Nutritional Knowledge of the women was gauged through 16 nutritional knowledge questions on the feature of nutrition needed in their course of pregnancy. Therefore, nutritional knowledge score was computed by conducting principal component analysis to reduce data and

identify nutritional knowledge. Then, summing the factor scores into one composite and ranking into terciles (three parts) was performed. Lastly, the highest tercile was defined as having "Good" nutritional knowledge and the two lower tercile were labeled as "Poor" nutritional knowledge.

Maternal attitude was evaluated with 12 Likert scale questions using PCA. The factor scores were totaled and classified into terciles (three parts), and the highest tercile was defined as having "Favorable" maternal attitude and the two lower tercile were characterized as "Unfavorable" maternal attitude. In similar way, perceived vulnerability, perceived severity to malnutrition, perceived benefit to healthy nutrition, perceived barrier to healthy nutrition and perceived self-efficacy to control malnutrition during pregnancy were calculated by using their composite questions. Finally, the total scores were converted into terciles and the highest terciles were used to labeled as women were "having" perceived vulnerability, perceived severity to malnutrition, perceived benefit to healthy nutrition, perceived barrier to healthy nutrition and perceived self-efficacy to control malnutrition, else no if with both lower terciles. Women's autonomy was evaluated by seven validated questions which were adopted from Ethiopian demographic health survey (28). For each response of a question, the response to each question was coded as "one" when decision was made by the pregnant women alone or jointly with their husband, otherwise "zero".

Ethical consideration

This study was conducted in agreement with the Declaration of Helsinki-Ethical principle for medical research involving the human subjects. Accordingly, ethical approval letter was obtained from Haramaya University Institutional Research Ethics and Review Committee (IRERC) with a reference number of (IHRERC/266/2020) before commencement of data collection. Informed, voluntarily, written and signed consent was obtained from study participants and privacy and confidentiality was maintained. An official letter was sent to Haramaya district health office and respective kebeles. The personal identifiers were excluded, information gained was kept confidential and used for the proposed study only.

Operational definition

Dietary diversity: Is the number of foods consumed across and within food groups over seven day reference period.

Dietary diversity score: Is the sum of each of food groups the pregnant women consumed over period of week

Meal frequency: is defined as how many times a day peoples eat or a number of daily eating occasion

Appropriate dietary practice: when women had at least four meals daily, good FVS, high DDS, and high ASF consumption, whereas it was inappropriate when women had less than four meals daily or Low FVS or low DDS or low ASF consumption (23)

High DDS: Tercile calculated from food groups, and the highest tercile was considered as a high DDS, whereas the rest two lower terciles were taken as a low DDS .

High DDS: terciles were calculated from food groups, and the highest tercile was considered as a high DDS, whereas the rest two lower terciles were taken as low DDS (25)

Nutritional Knowledge: Was measured through16 nutritional knowledge questions on the feature of nutrition needed in pregnancy and the score was computed by conducting PCA. Then composite was ranked into terciles (20)

Good nutritional knowledge: Tercile computed from nutritional knowledge and the highest tercile was defined as good nutritional knowledge whereas, the two lower tercile were labeled as Poor

Wealth index quintile: Was computed from wealth score of the households by PCA and composite was ranked into quantile. Quintile was used to label the households wealth status to poorest, poor, middle, rich and richest category

Result

Socio-demographic and obstetric characteristics of pregnant women

The socio-demographic and obstetric characteristics of the respondent were shown in (table 1). In this study four hundred forty-eight pregnant women were participated, making a response rate of 99.5%. The mean (± SD) age of women were 25.68(± 5.16) years. Almost more than three-fourth of pregnant women had no formal education. The wealth index displayed that 19.87% of the respondent were in the richest class. Only 60.49% of the households have land for agriculture

Table 1: Socio-demographic and obstetric characteristics of pregnant women in Haramaya district, eastern Ethiopia, 2021

Variables	Frequency(n=448)	Percentage (%)
Age Category of Pregnant women (PW)		

15-17	25	5.58
18-24	166	37.05
25-34	234	52.23
$\geq$35	23	5.13
Mean ($\pm$ SD)	25.68($\pm$5.16)	
Educational level of women		
Can't read and write	331	73.88
Informal education	26	5.81
Formal education^{+}	91	20.31
Educational Level of Husband		
Can't read and write	259	57.81
Can read and write	61	13.62
Elementary School (Grade 1-8)	102	22.77
High school and above	26	5.8
Occupational status of Women		
House wife	433	96.65
Merchants*	15	3.65
Occupational status of Husband		
Farmer	420	93.75
Merchant*	28	6.25
Family size		
1-5	343	76.56
>5	105	23.44
Agricultural land possession		
Yes	271	60.49
No	177	39.51
Livestock Possession		
Yes	299	66.74
No	149	33.26
Wealth Index (Quantile)		
Poorest	90	20.09
Poor	90	20.09
Middle	89	19.87
Rich	90	20.09
Richest	89	19.87
Stage of pregnancy		
First Trimester	19	4.24
Second trimester	296	66.07
Third trimester	133	29.69
ANC Visit		
Yes	164	36.61
No	284	63.39
Parity		
Zero	103	22.99
Para 1-4	294	65.63
Five and more	51	11.38
Gravidity		
Less than or equal five	375	83.71
Greater than five	73	16.29

PW: Pregnant Women; Merchant*: include petty trading; ANC: Antenatal Care
Housewife: Women whose activities are in home and not participated in yielding family financial income
Formal education^{+}: Refers grade 1 and above
Wealth index quintile: Was computed from wealth score of the households and used to label households wealth status to five categories
Family Size: Refers to total number of family members living together
Parity: Refers to the number of births the mother experienced after 28 weeks whatever the status of the newborn is (WHO,2015)

Prevalence of Dietary Practice

From total respondents 29.46% (0.25, 0.34), 37.5% mean ($\pm$ SD) (9.03 $\pm$ 2.79) and 24.78% (0.21, 0.29) of them had high dietary diversity, high food variety score and high consumption of animal source food respectively. The prevalence of appropriate dietary practice of pregnant women in present study was 15.18% (95% CI=11.96-18.84) (Fig 1). The major

consumed food groups were cereal (100%), Other vitamin A-rich fruits and vegetables (96.43%) and pulses and nuts (53.35%) food groups. Consumption of other fruits and other vegetables were minimal (Fig 2).

Factors Associated with Dietary Practice

Multivariable analysis was applied to identify factors associated with dietary practice of pregnant women using Poisson regression analysis model with robust variance estimate. From this analysis the prevalence of dietary practice was higher among merchant women (APR=2.07; 95% CI 1.071- 4.016 and those whose husband have high school and above educational level (APR=1.96; 95% CI=1.1.063-3.460) .However, the prevalence of dietary practice was significantly lower among those who reported chat chewing (APR=0.58; 95% CI= 0.370- 0.901) and avoiding food intake (APR= 0 .36; 95% CI=0.200- 0.651) (table)

Variables	Dietary Practice Inappropriate (n=380)	Dietary Practice App[illegible]iate (n=68)	CPR (95%CI)	APR (95%CI)	P-value
Educational Level of Husband					
Illiterate	287 (75.53)	33(48.53)		1	
Elementar[illegible]hool	*76 (20.00)*	26(38.24)	2.47(1.554, 3.929)	1.99 (1.214, 3.280)	0.00
High school and above	17(4.47)	9(13.24)	3.37(1.806, 6.239)	**1.92 (1.063, 3.460)**	**6**
Occupational status of Women					
House wife	372(97.89)	61(89.71)		1	
Others	8 (2.11)	7(10.29)	3.31(1.837, 5.973)	**2.07 (1.071, 4.016)**	**0.030**
ANC visit					
No	147 (38.6)	17(25.00)		1	
Yes	233(61.32)	51(75.00)	1.73(1.035, 2.898)	1.36 (0.776 ,2.394)	0.281
Perceived Venerability					
No	313(82.37)	50(73.53)		1	
Yes	67 (17.63)	18(26.47 |)	1.537412 (0.947, 2.496)	0.82 (.469 1.428	0.481
Perceived severity					
No	313 (82.37)	47(69.12)		1	
Yes	67 (17.63)	21(30.88)	1.827853 (1.155, 2.892)	1.31 (0.747 2.303)	0.346
Perceived Benefit					
No	288 (75.79)	39(57.35)		1	
Yes	92 (24.21)	29(42.65)	2.01(1.302, 3.099)	1.39 (0.811, 2.412)	0.228
Chat chewing					
No	142 (37.37)	38(55.88)		1	
Yes	238 (62.63)	30(44.12 |)	.5302435 (0.341, 0.824)	**0.58 (0.370, 0.901)**	**0.016**
Drinking water					
Protected	156 (41.05)	42(61.76)		1	
Unprotected	224 (58.95)	26 (38.24)	0.49(0.312,0.771)	0.61(0.350, 1.048)	0.073
Avoiding Food taking					
No	246(64.74)	53(77.94)			
Yes	134(35.26)	15(22.06)	0.57 (0.331, 0.974)	**0.36(0.200, 0.651)**	**0.001**
Food aversion (Strong disliked of foods)					
No	113(29.74)	21(30.88)		1	
yes	267 (70.26)	47(69.12)	0.96(0.594, 1.533)	0.99 (0.631, 1.554)	0.967

Appropriate dietary practice: is defined as the consumption of at least four meals daily, high DDS, high FVS and high ASF; otherwise, Inappropriate dietary practice
Elementary school: Grade 1-8
CPR= Crude Prevalence Ration; APR= Adjusted Prevalence Ratio, CI= Confidence Interval at 95%
CPR: was obtained from bi-variate of Poisson regression analysis model with robust variance estimate
APR, CI and P-Value were found from multivariable Poisson regression analysis model with robust variance estimate

Table 2: Factors associated with dietary practice among pregnant women in Haramaya district, Eastern Ethiopia,2021

Discussion

Adequate nutrition in pregnancy is important for maternal and child health (3). There is mounting evidences that, insufficient consumption of balanced and quality diet during pregnancy has a great effect on fetus health and development and may also result to poor birth outcome (4). At beginning of pregnancy many women lack sufficient micronutrients stores to meet physiological requirement (1) and they are more vulnerable to malnutrition (29). Several epidemiological studies indicated inappropriate dietary practice is significant contributor to maternal under nutrition and micronutrients deficiency in resource limited countries (30). Therefore, the diet of pregnant women ought to supply an adequate quantity of nutrients for mother, fetus and for effective lactation . Despite this reality, in the current study only 15.18% (95% CI=11.96-18.84) of pregnant women had appropriate dietary practice and noted to be very much low. This figure is very much lower than studies conducted in Illu Aba Bor Zone, Southwest Ethiopia (31) , Dessie town, northeastern Ethiopia (20) and Guto Gida district, western Ethiopia (32). The result of this study has modest deference with study conducted in Gojjam in northwest Ethiopian (19.9%) (6) , which was carried out on similar setup/rural residents. However, due to the differences in study area and socio-culturally conditions, it is noteworthy to memo that the direct comparison of our result with previous investigations employed in Ethiopia is impossible. Furthermore, the possible reason for the discrepancy might be due to the deference in methods and measures applied to assess dietary practice since this study used four measures; DDS, FVS, consumption of ASF and frequency of meal over a period of one week. However, most of the previous studies carried out in Ethiopia used meal frequency to evaluate dietary practice of pregnant women which could not comprehensively assess dietary practice.

Dietary diversity is correlated with the probability of nutrient adequacy, increased nutrient intake and better nutritional status of pregnant women (33) Evidences suggested that consumption of animal source foods guarantees intake of micronutrients among pregnant women in developing countries (22, 34). Though, consumption of animal source food has significant share to dietary quality, the practice is decreasing in low-income counties (35). In the present study, we observed that only 29.46%, of pregnant women have high dietary diversity score. However, 37.5% of participants have high mean food variety score. Furthermore, 24.78% of women consumed animal source foods over a period of one-week prior the survey. The current finding of dietary diversity in pregnancy is comparably lower than various studies conducted in Ethiopia (5,20,31-32), but higher than study carried out in Shashemane town, central Ethiopia (36).

Similar to reports from shashamane (36) and Egypt (37), our results also display that occupational status of pregnant women was found to have a significant association with dietary practice. Pregnant women who were merchants had higher dietary practice. Housewife women are found to be confined more to household works that have devalued financial value to and, are more rely on their family and their partner as they are compared to those women who engaged in yielding their family financial gain Nepal (38). This is could be because of women who participated in yielding their family financial income have a better chance of earning income and consequently a better access to diversified foods and appropriate diet. This suggests that program to improve women nutrition would strength on increasing women involvement in family income generation, decision making power and their economic independency .

The current study also presented that women who have partners of high school and above educational level had higher appropriate dietary practice, which is consistent to results of the former studies done in wachamo (39) Shashamane (36). This might be due to the reality that literate partners have better understanding about the importance of consuming quality diet in pregnancy and could positively influence their wife to have appropriate dietary practice. Pregnant women who received dietary guidance are more expected to intake diversified diet than those women who do not received nutritional guidance

In this study, diet of pregnant women who were frequently involved in avoiding the intake of food was negatively associated with appropriate dietary practice which is inline with study carried out in Nepal (40) and China (41) . Pregnant women practiced food avoiding have higher odd of insufficient dietary practice. Our result may indicate that the practice of food avoidance could lead to declined dietary consumption and then micronutrient deficiency. The possible rationale of the deference might be mainly in the present study most of the pregnant women who did avoiding of food intake did so in the third trimester of their pregnancy and at the time of interview

Although, chewing khat is a intensively disseminating act in Ethiopia and in developed countries , comprising America and Europe (42-44) its consequence on health is not so far richly understood. The result of the current study recommends the importance of reducing the high level of inappropriate dietary practice during pregnancy with the proper intervention in the related discourse factors. Pregnant women should be advised the negative consequence of chat chewing frequently and supported to rise their dietary consumption in pregnancy .

Validated Food frequency questionnaires was used to asses dietary practice. Food items was established based on wide-ranging consultation of key informants who are from the study area and who knew the culture and local language on types and food groups usually consumed, which is the strength of this study. Various limitations required to be considered when interpreting our results. The cross-sectional nature of the data limits causal inference between dietary practice and the correspond. Since there is within individual difference of dietary consumption in the study setup over the period of seven days, we establish our definition the reference time period of seven day. Women who could have eaten a food items more than one in seven day were also tagged with those who consumed one time over seven day could underrate the amount used-up is another limitation . The cross-sectional nature of the data limits causal inference between dietary diversity and the correlates. Also confinement of sample to a single season may limit the generalizability of result to other seasons.

Conclusion

The study showed that dietary practice of pregnant women in Haramaya district is sub-optimal. It can be concluded that greater access to resources through: involvement of women in yielding their family financial income, partners education , dietary restriction and chewing chat , as gauged by the study, was noted to be significantly associated with dietary practice during pregnancy. Thus we suggest nutrition policy, programs and interventions should aimed at encouraging prenatal dietary practice focusing on women empowerment in generating their family income and raising partners awareness on the benefit of quality diet in pregnancy both for the mother and the newborn. Partners nutritional counselling and intervention should be tailored to meet the need of pregnant women and to improve their dietary practice. To reduce the habit of chat chewing during pregnancy dietary guidance and counselling are suggested.

Abbreviations

ANC, Antenatal Care; ASF, Animal Source Food; DDS, Dietary Diversity Score; EDHS, Ethiopian Demographic and Health Survey; FVS, Food Variety Score; HDS-HRC, Health Demographic Surveillance and Health Research Center; PCA, Principal Component Analysis; WHO, World Health Organization

Consent for publication

Not applicable

Competing interests

The authors declare no conflicts of interest in this work.

Authors' contributions

All authors made momentous involvement to the conception, design of the study, and statistical analysis and result interpretation. MBF, KTR, LO, AKT and TAY drafted the manuscript, reviewed critically, revised the work briefly, gave final approval of the version to be published and agreed to be accountable for all aspects of the article

Funding

This study was fully funded by Haramaya University after the proposal has been defended. The funder has no role in conception, design of the study, statistical analysis, result interpretation and in writing up the manuscript. The funding institution has no role in the publication consent or approval.

Acknowledgment

The authors would like express sincere appreciation to Haramaya University for funding of this study. Special thanks go to Haramaya district health office staffs for their enormous support in the whole period of data collection. We acknowledge all study subjects volunteered for the study, data collectors and supervisors for their cooperation and meticulous contributions.

References

1. KRAMER, M. S. 1998. Maternal nutrition, pregnancy outcome and public health policy. *CMAJ: Canadian Medical Association Journal,* 159**,** 663.
2. FIONA BROUGHTON PIPKIN MA, D., FRCOG AD EUNDEM 2012. Maternal Physiology. *Dewhurst's Textbook of Obstetrics & Gynaecology.*
3. KRAMER, M. S. 1987. Determinants of low birth weight: methodological assessment and meta-analysis. *Bulletin of the world health organization,* 65**,** 663.
4. GOGOI, M. & PRUSTY, R. K. 2013. Maternal anaemia, pregnancy complications and birth outcome: evidences from north-east India. *Journal of North East India Studies,* 3**,** 74-85.
5. ALEMAYEHU, M. S. & TESEMA, E. M. 2015. Dietary practice and associated factors among pregnant women in Gondar town north west, Ethiopia, 2014. *Int J Nutr Food Sci,* 4**,** 707-12.

6. DEMILEW, Y. M., ALENE, G. D. & BELACHEW, T. 2020. Dietary practices and associated factors among pregnant women in West Gojjam Zone, Northwest Ethiopia. *BMC Pregnancy and Childbirth,* 20**,** 18.
7. LEYNA, G. H., MMBAGA, E. J., MNYIKA, K. S., HUSSAIN, A. & KLEPP, K.-I. 2010. Food insecurity is associated with food consumption patterns and anthropometric measures but not serum micronutrient levels in adults in rural Tanzania. *Public Health Nutrition,* 13**,** 1438-1444.
8. OLDEWAGE-THERON, W. H. & EGAL, A. A. 2010. Nutrition knowledge and nutritional status of primary school children in QwaQwa. *South African Journal of Clinical Nutrition,* 23.
9. LEE, S. E., TALEGAWKAR, S. A., MERIALDI, M. & CAULFIELD, L. E. 2013. Dietary intakes of women during pregnancy in low-and middle-income countries. *Public health nutrition,* 16**,** 1340-135
10. LANDER, R. L., HAMBIDGE, K. M., WESTCOTT, J. E., TEJEDA, G., DIBA, T. S., MASTIHOLI, S. C., ET AL 2019. . Pregnant women in four low-middle income countries have a high prevalence of inadequate dietary intakes that are improved by dietary diversity *Nutrients,* 11**,** 1560.
11. OTHOO, D., WAUDO, J. & KURIA, E. 2014. Dietary assessment of vitamin A and Iron among pregnant women at Ndhiwa Sub District Hospital–Kenya. *African Journal of Food, Agriculture, Nutrition and Development,* 14**,** 2114-2128.
12. KENNEDY, G., FANOU-FOGNY, N., SEGHIERI, C., ARIMOND, M., KOREISSI, Y., DOSSA, R., et al. 2010. Food groups associated with a composite measure of probability of adequate intake of 11 micronutrients in the diets of women in urban Mali. *The Journal of nutrition,* 140**,** 2070S-2078S.

13. KENNEDY, G. L., PEDRO, M. R., SEGHIERI, C., NANTEL, G. & BROUWER, I. 2007. Dietary diversity score is a useful indicator of micronutrient intake in non-breast-feeding Filipino children. *The Journal of nutrition,* 137**,** 472-477.
14. OGLE, B. M., HUNG, P. H. & TUYET, H. T. 2001. Significance of wild vegetables in micronutrient intakes of women in Vietnam: an analysis of food variety. *Asia Pacific journal of clinical nutrition,* 10**,** 21-30.
15. HOP, L. T. 2003. Programs to improve production and consumption of animal source foods and malnutrition in Vietnam. *The journal of nutrition,* 133**,** 4006S-4009S.
16. MARSHALL, T. A., STUMBO, P. J., WARREN, J. J. & XIE, X.-J. 2001. Inadequate nutrient intakes are common and are associated with low diet variety in rural, community-dwelling elderly. *The Journal of nutrition,* 131**,** 2192-2196.
17. NEUMANN, C. & HARRIS, D. M. 1995. Contribution of animal source foods in improving diet quality for children in the developing world

18. LINDSAY, K., GIBNEY, E. & MCAULIFFE, F. 2012. Maternal nutrition among women from Sub-Saharan Africa, with a focus on Nigeria, and potential implications for pregnancy outcomes among immigrant populations in developed countries. *Journal of human nutrition and dietetics,* 25**,** 534-546.
18. Haramya District Health Office. Annual report of Haramya district Health Office 2020. Haramya, Eastern Ethiopia; 2020
19. NANA, A. & ZEMA, T. 2018. Dietary practices and associated factors during pregnancy in northwestern Ethiopia. *BMC pregnancy and childbirth,* 18**,** 183.
20. KENNEDY, G., BALLARD, T. & DOP, M. C. 2011. *Guidelines for measuring household and individual dietary diversity*, Food and Agriculture Organization of the United Nation
21. ALLEN, L. H. 2003. Interventions for micronutrient deficiency control in developing countries: past, present and future. *The Journal of nutrition,* 133**,** 3875S-3878S.
22. BELACHEW, T., LINDSTROM, D., GEBREMARIAM, A., HOGAN, D., LACHAT, C., HUYBREGTS, L., et al. 2013. Food insecurity, food based coping strategies and suboptimal dietary practices of adolescents in Jimma zone Southwest Ethiopia. *PloS one,* 8**,** e57643.
23. GEBREYESUS, S. H., LUNDE, T., MARIAM, D. H., WOLDEHANNA, T. & LINDTJØRN, B. 2015. Is the adapted Household Food Insecurity Access Scale (HFIAS) developed internationally to measure food insecurity valid in urban and rural households of Ethiopia? *BMC Nutrition,* 1**,** 1-10

24. WORKICHO, A., BELACHEW, T., FEYISSA, G. T., WONDAFRASH, B., LACHAT, C., VERSTRAETEN, R., et al. 2016. Household dietary diversity and Animal Source Food consumption in Ethiopia: evidence from the 2011 Welfare Monitoring Survey. *BMC public health,* 16**,** 1-11.
25. KANT, A. K. & THOMPSON, F. E. 1997. Measures of overall diet quality from a food frequency questionnaire: National Health Interview Survey, 1992. *Nutrition Research,* 17**,** 1443-1456.
26. TORHEIM, L. E., BARIKMO, I., PARR, C. L., HATLØY, A., OUATTARA, F. & OSHAUG, A. 2003. Validation of food variety as an indicator of diet quality assessed with a food frequency questionnaire for Western Mali. *European Journal of Clinical Nutrition,* 57**,** 1283-1291.
27. Central Statistical Agency(CSA)(Ethiopia) and ICF. Ethiopia demographic and health survey 2016. Addis Ababa and Rockville: CSA and ICF; 2016
28. SUKRAT, B. 2014. Thailand adolescent birth rate: trend and related indicators. *Thai Journal of Obstetrics and Gynaecology***,** 15-21.
29. HOFFMANN, J. F., NUNES, M. A. A., SCHMIDT, M. I., OLINTO, M. T. A., MELERE, C., OZCARIZ, S. G. I., et al. 2013. Dietary patterns during pregnancy and the association with sociodemographic characteristics among women attending general practices in southern Brazil: the ECCAGe Study. *Cadernos de saude publica,* 29**,** 970-980.
30. TSEGAYE, D., TAMIRU, D. & BELACHEW, T. 2020. Factors Associated with Dietary Practice and Nutritional Status of Pregnant Women in Rural Communities of Illu Aba Bor Zone, Southwest Ethiopia. *Nutrition and Dietary Supplements,* 12**,** 103-112.
31. DABA, G., BEYENE, F., FEKADU, H. & GAROMA, W. 2013. Assessment of knowledge of pregnant mothers on maternal nutrition and associated factors in Guto Gida Woreda, East Wollega Zone, Ethiopia. *Journal of Nutrition & Food Sciences,* 3**,** 1.
32. MIRMIRAN, P., ESMAILLZADEH, A. & AZIZI, F. 2004. Detection of cardiovascular risk factors by anthropometric measures in Tehranian adults: receiver operating characteristic (ROC) curve analysis. *European journal of clinical nutrition,* 58**,** 1110-1118.
33. STEYN, N. P., NEL, J. H., NANTEL, G., KENNEDY, G. & LABADARIOS, D. 2006. Food variety and dietary diversity scores in children: are they good indicators of dietary adequacy? *Public health nutrition,* 9**,** 644-650.
34. SPEEDY, A. W. 2003. Global production and consumption of animal source foods. *The Journal of nutrition,* 133**,** 4048S-4053S.
35. DESTA, M., AKIBU, M., TADESE, M. & TESFAYE, M. 2019. Dietary Diversity and Associated Factors among Pregnant Women Attending Antenatal Clinic in Shashemane, Oromia, Central Ethiopia: A Cross-Sectional Study. *Journal of nutrition and metabolism,* 2019.
36. ALKALASH, S. H., HEGAZY, N. N., ELNADY, R. T. & KHALIL, N. A. 2021. Dietary Practice and Nutritional Status Among Pregnant Women. *The Egyptian Journal of Hospital Medicine,* 83**,** 1030-1037
38. SHRESTHA, V., PAUDEL, R., SUNUWAR, D. R., LYMAN, A. L. T., MANOHAR, S. & AMATYA, A. 2021. Factors associated with dietary diversity among pregnant women in the western hill region of Nepal: A community based cross-sectional study. *PloS one,* 16**,** e0247085.
39 . DELIL, R., ZINAB, B., MOSA, H., AHMED, R. & HASSEN, H. 2021. Determinants of dietary diversity practice among pregnant women attending antenatal clinic at Wachemo University Nigist Eleni Mohammed memorial referral hospital, Southern Ethiopia. *PloS one,* 16**,** e0250037
40. LAMINA, S. 2010. Khat (Catha edulis): the herb with officio-legal, socio-cultural and economic uncertainty. *South African Journal of Science,* 106**,** 1-4..

41. GAO, H., STILLER, C. K., SCHERBAUM, V., BIESALSKI, H. K., WANG, Q., HORMANN, E., et al. 2013. Dietary intake and food habits of pregnant women residing in urban and rural areas of Deyang City, Sichuan Province, China. *Nutrients,* 5**,** 2933-2954.

42. GEBISSA, E. 2010. Khat in the Horn of Africa: Historical perspectives and current trends. *Journal of ethnopharmacology,* 132**,** 607-614.

GRIFFITHS, P., LOPEZ, D., SEDEFOV, R., GALLEGOS, A., HUGHES, B., NOOR, A., et al. 2010

43. Khat use and monitoring drug use in Europe: the current situation and issues for the future. *Journal of ethnopharmacology,* 132**,** 578-583.
44. ODENWALD, M., WARFA, N., BHUI, K. & ELBERT, T. 2010. The stimulant khat—another door in the wall? A call for overcoming the barriers. *Journal of Ethnopharmacology,* 132**,** 615-619.

Figure

Click here to access/download;Figure;Figure File of dietary Practice.docx

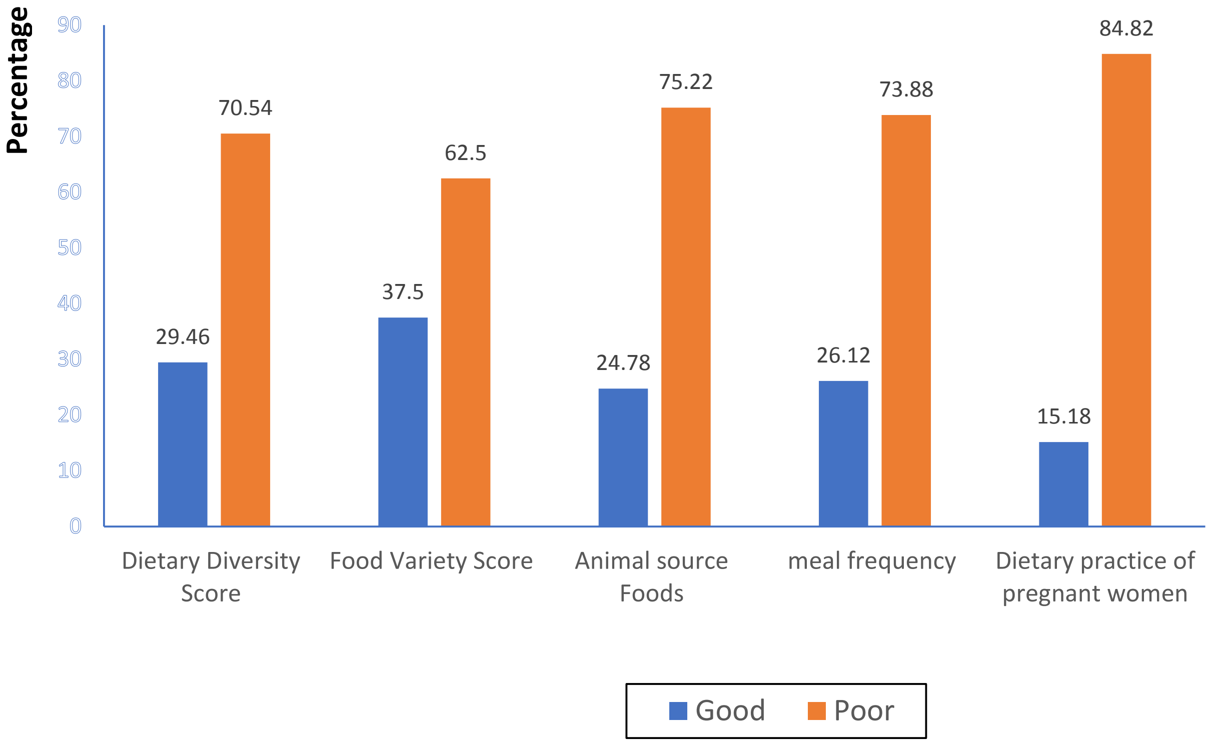

Figure 1: Dietary practices of pregnant women in Haramaya district, eastern Ethiopia, 2021

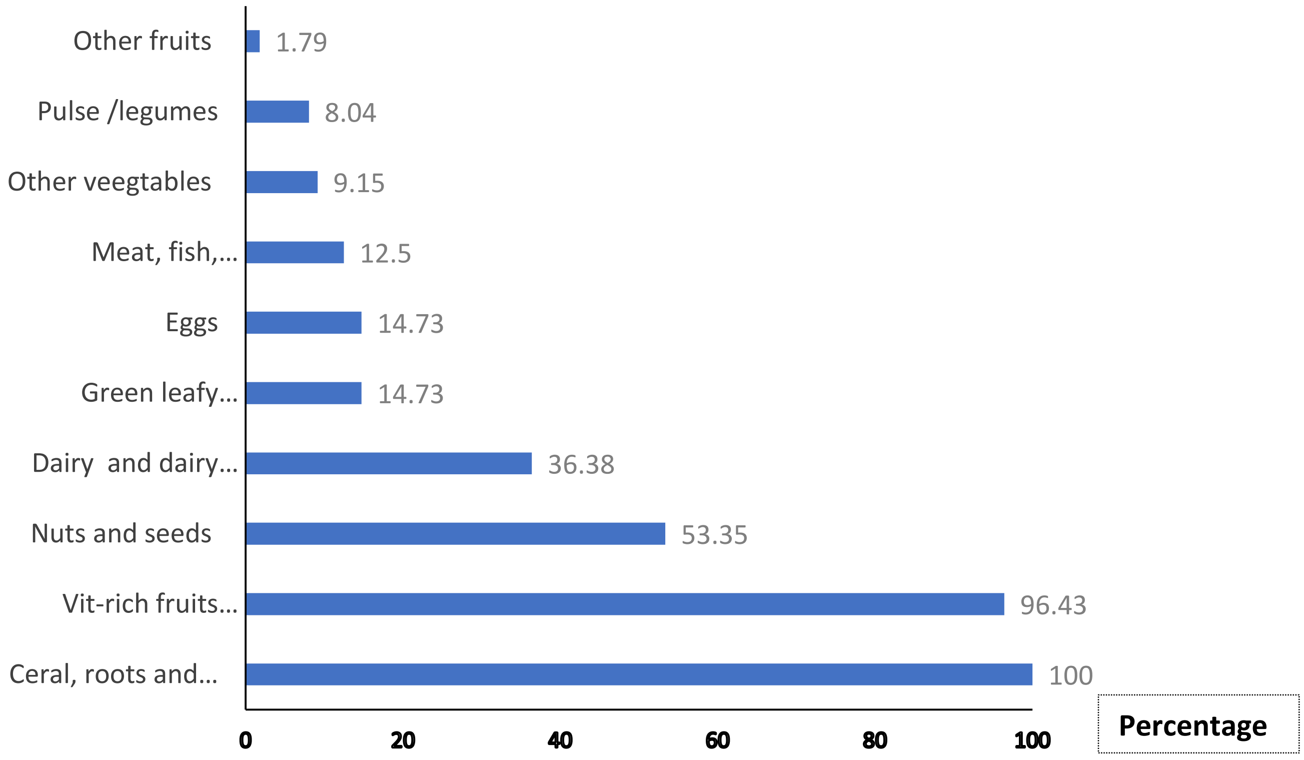

Figure 2: Percentage of different food groups consumed by pregnant women in Haramaya district, eastern Ethiopia, 2021
