# Supplementary material for: Consumption of animal source food and associated factors among pregnant women in eastern Ethiopia: A community-based study
Source: PLoS One. 2022 Jun 17;17(6):e0270250. doi: 10.1371/journal.pone.0270250 (PMC9205500; doi:10.1371/journal.pone.0270250)
Supplement: Attachment — Submitted filename: Response to Reviewers.docx [file pone.0270250.s002.docx]

AUTHORS’ RESPONSES TO THE EDITORS AND REVIEWERS’ COMMENTS AND SUGGESTIONS

| **PLOS ONE** <em@editorialmanager.com> | Dec 14, 2021, 9:45 AM |  |    |
| --- | --- | --- | --- |
| to me  | | |  |

PONE-D-21-17077

Dietary Practices and Associated Factors Among Pregnant Women in Haramaya District, Eastern Ethiopia

PLOS ONE

Dear Lucinda Shen!

Staff Editor
PLOS ONE

First of all we would like to acknowledge you for your constructive and valuable comments and helpful
suggestions that helped us to improve and enrich our manuscript. Here under following editors comment and each reviewer’s we have pointed out how authors incorporated your valuable comments, suggestions and concerns one by one. On the following pages, you will find our response to the editor and reviewer comments. On behalf of my co-authors, I thank you for your consideration of this re-submission.

Meseret Belete Fite, meseretphd2014@gmail.com (correspondence author)

Editor comments and authors’ response

Thank you for submitting your manuscript to PLOS ONE. After careful consideration, we feel that it has merit but does not fully meet PLOS ONE’s publication criteria as it currently stands. Therefore, we invite you to submit a revised version of the manuscript that addresses the points raised during the review process.

The manuscript has been evaluated by three reviewers, and their comments are available below.

The reviewers have raised a number of major concerns to the study methodology. They feel that considerations of potential confounding variables should be accounted for in the statistical technique used; and the introduction could be further expanded to describe how the current study further contributes to scientific knowledge in light of the articles which the reviewer has noted.

Thank you a lot for your substantial effort. Really this comment is helpful to enrich the manuscript. Authors get your concern and critically looked your comment.

Considering the reviewer #3 has argued that“Meta-analysis was conducted on the dietary practices ” and suggested us to modify the research title to generate new evidence for scientific community and nutrition experts, we have tied to modify the research tittle to consumption of animal source food and associated factors among pregnant women in Eastern Ethiopia: A community-based study.

- We have also tied to consider all the comments and suggestions raised by the reviewers and incorporated in the revised manuscript.

- Major modification of title was made

- Major revision of methodology and statistical model of analysis was done. We have changed statistical statistical model of analysis from Poisson regression analysis model with robust variance estimate to binary logistic regression model

- We have tied to re-write introduction and discussion

Finally, they feel that the copy editing is needed on the manuscript. Please note that one of the publication criteria at PLOS ONE ([https://journals.plos.org/plosone/s/criteria-for-publication#loc-5](https://journals.plos.org/plosone/s/criteria-for-publication#loc-5) )is that articles must be presented in an intelligible fashion and written in clear, correct, and unambiguous English

Could you please carefully revise the manuscript to address all comments raised?

**Authors’ responses:** Thank you very much. We appreciated your critical and thorough review of our manuscript and constructive comments that will improve our paper to its best. We have tried to correct typos and grammatical errors in the revised manuscript using language expert.

Please submit your revised manuscript by Jan 20 2022 11:59PM. If you will need more time than this to complete your revisions, please reply to this message or contact the journal office at [plosone@plos.org](mailto:plosone@plos.org). When you're ready to submit your revision, log on to [https://www.editorialmanager.com/pone/](https://www.editorialmanager.com/pone/) and select the 'Submissions Needing Revision' folder to locate your manuscript file.

Please include the following items when submitting your revised manuscript:

- A rebuttal letter that responds to each point raised by the academic editor and reviewer(s). You should upload this letter as a separate file labeled 'Response to Reviewers'.
- A marked-up copy of your manuscript that highlights changes made to the original version. You should upload this as a separate file labeled 'Revised Manuscript with Track Changes'.
- An unmarked version of your revised paper without tracked changes. You should upload this as a separate file labeled 'Manuscript'.

**Authors’ responses:** Thank you very much. We appreciated submitted a rebuttal letter that responds to each point raised by the academic editor and reviewer(s)

We have marked-up copy of your manuscript that highlights changes made to the original version.

If you would like to make changes to your financial disclosure, please include your updated statement in your cover letter. Guidelines for resubmitting your figure files are available below the reviewer comments at the end of this letter.

If applicable, we recommend that you deposit your laboratory protocols in [protocols.io](http://protocols.io/) to enhance the reproducibility of your results. Protocols.io assigns your protocol its own identifier (DOI) so that it can be cited independently in the future. For instructions see: [https://journals.plos.org/plosone/s/submission-guidelines#loc-laboratory-protocols](https://journals.plos.org/plosone/s/submission-guidelines#loc-laboratory-protocols). Additionally, PLOS ONE offers an option for publishing peer-reviewed Lab Protocol articles, which describe protocols hosted on [protocols.io](http://protocols.io/). Read more information on sharing protocols at [https://plos.org/protocols?utm_medium=editorial-email&utm_source=authorletters&utm_campaign=protocols](https://plos.org/protocols?utm_medium=editorial-email&utm_source=authorletters&utm_campaign=protocols).

2. Please include additional information regarding the survey or questionnaire used in the study and ensure that you have provided sufficient details that others could replicate the analyses. For instance, if you developed a questionnaire as part of this study and it is not under a copyright more restrictive than CC-BY, please include a copy, in both the original language and English, as Supporting Information. If the original language is written in non-Latin characters, for example Amharic, Chinese, or Korean, please use a file format that ensures these characters are visible.

3. Thank you for stating the following in the Acknowledgments Section of your manuscript:

“The authors would like express sincere appreciation to Haramaya University for funding of this study. Special thanks go to Haramaya district health office staffs for their enormous support in the whole period of data collection.  We acknowledge all study subjects volunteered for the study, data collectors and supervisors for their cooperation and meticulous contributions. “

We note that you have provided additional information within the Acknowledgements Section that is not currently declared in your Funding Statement. Please note that funding information should not appear in the Acknowledgments section or other areas of your manuscript. We will only publish funding information present in the Funding Statement section of the online submission form.

Please remove any funding-related text from the manuscript and let us know how you would like to update your Funding Statement. Currently, your Funding Statement reads as follows:

“This study was fully funded by Haramaya University, Ethiopia. The funder has no role in conception, design of the study, statistical analysis, result interpretation and in writing up the manuscript. The funding institution has no role in the publication consent or approval.”

Please include your amended statements within your cover letter; we will change the online submission form on your behalf.

4. In your Data Availability statement, you have not specified where the minimal data set underlying the results described in your manuscript can be found. PLOS defines a study's minimal data set as the underlying data used to reach the conclusions drawn in the manuscript and any additional data required to replicate the reported study findings in their entirety. All PLOS journals require that the minimal data set be made fully available. For more information about our data policy, please see [http://journals.plos.org/plosone/s/data-availability](http://journals.plos.org/plosone/s/data-availability).

"Upon re-submitting your revised manuscript, please upload your study’s minimal underlying data set as either Supporting Information files or to a stable, public repository and include the relevant URLs, DOIs, or accession numbers within your revised cover letter. For a list of acceptable repositories, please see [http://journals.plos.org/plosone/s/data-availability#loc-recommended-repositories](http://journals.plos.org/plosone/s/data-availability#loc-recommended-repositories). Any potentially identifying patient information must be fully anonymized.

Important: If there are ethical or legal restrictions to sharing your data publicly, please explain these restrictions in detail. Please see our guidelines for more information on what we consider unacceptable restrictions to publicly sharing data: [http://journals.plos.org/plosone/s/data-availability#loc-unacceptable-data-access-restrictions](http://journals.plos.org/plosone/s/data-availability#loc-unacceptable-data-access-restrictions). Note that it is not acceptable for the authors to be the sole named individuals responsible for ensuring data access.

We will update your Data Availability statement to reflect the information you provide in your cover letter.

5. Please include a separate caption for each figure in your manuscript

[Note: HTML markup is below. Please do not edit.]

Reviewers' comments:

**Reviewer's comments and authors’ response**

**Comments to the Author**

1. Is the manuscript technically sound, and do the data support the conclusions?

The manuscript must describe a technically sound piece of scientific research with data that supports the conclusions. Experiments must have been conducted rigorously, with appropriate controls, replication, and sample sizes. The conclusions must be drawn appropriately based on the data presented.

Reviewer #1: Yes

Reviewer #2: Yes

Reviewer #3: No

2. Has the statistical analysis been performed appropriately and rigorously?

Reviewer #1: No

Reviewer #2: Yes

Reviewer #3: Yes

3. Have the authors made all data underlying the findings in their manuscript fully available?

The [PLOS Data policy](http://www.plosone.org/static/policies.action#sharing) requires authors to make all data underlying the findings described in their manuscript fully available without restriction, with rare exception (please refer to the Data Availability Statement in the manuscript PDF file). The data should be provided as part of the manuscript or its supporting information, or deposited to a public repository. For example, in addition to summary statistics, the data points behind means, medians and variance measures should be available. If there are restrictions on publicly sharing data—e.g. participant privacy or use of data from a third party—those must be specified.

Reviewer #1: Yes

Reviewer #2: Yes

Reviewer #3: Yes

4. Is the manuscript presented in an intelligible fashion and written in standard English?

PLOS ONE does not copyedit accepted manuscripts, so the language in submitted articles must be clear, correct, and unambiguous. Any typographical or grammatical errors should be corrected at revision, so please note any specific errors here.

Reviewer #1: Yes

Reviewer #2: No

Reviewer #3: No

5. Review Comments to the Author

Please use the space provided to explain your answers to the questions above. You may also include additional comments for the author, including concerns about dual publication, research ethics, or publication ethics. (Please upload your review as an attachment if it exceeds 20,000 characters)

Reviewer #1:

Thank you a lot for your substantial effort. Really this comment is helpful to enrich the manuscript. Authors get your concern and critically looked your comment.

Considering the reviewer #3 has argued that“Meta-analysis was conducted on the dietary practices ” and suggested us to modify the research title to generate new evidence for scientific community and nutrition experts, we have tied to modify the research tittle to consumption of animal source food and associated factors among pregnant women in Eastern Ethiopia: A community-based study.

Additionally, this research is one objective of PhD dissertation work which was entitled “Prenatal Iron Status and Infants’ Birth Weight: Determinants of dietary practices, risk factors of iron deficiency, and infants’ birth weight and its association with maternal iron status in Haramaya district, eastern Ethiopia” by Meseret Belete Fite under supervention of the co-authored professors.

In this revised manuscript we have modified the title to “Consumption of animal source food and associated factors among pregnant women in Eastern Ethiopia: A community-based study.

For your information these are specific objectives the dissertation:

Specific objectives

1. To assess consumption of animal source food and associated factors among pregnant women in Eastern Ethiopia: A community-based study (Paper I)
2. To determine iron status and risk factors of iron deficiency among pregnant women in Haramaya district, eastern Ethiopia, 2021 (paper II)
3. Examine the associations between infants’ birth weight and maternal iron status in Haramaya district, eastern Ethiopia, 2021 (paper III)

Thus for this revised manuscript, we have two research question for our research . 1 what is the level of consumption of animal source food in haramya disrict. 2 what are the possible factors associated with consumption of animal source food. Actually all the detail of data for dietary practices of the pregnant women was collected as baseline for the cohort. However, as the the most common source of iron is animal source foods and we are interested to link the finding with the second objective(iron status and risk factors of iron deficiency) , we had to modified the the title . .

 Reviewer #1 comments

The regression model used by the authors does not seem appropriate. The outcome variable is binary.
The authors used Poisson regression which normally is used for count variables. The reason for the use of this model has not been clearly stated in the method section

Thank you so much. We appreciated your comments. We had fitted different analytical models and we have applied binary logistic regression model in revised manuscript. This model gave us narrow confidence interval , it was appropriate for our case.

However for your concern we wanted to give a brief why we used Poisson regression model in previous submission. We used in, Poisson regression analysis model with robust variance estimate for analysis previous submission . I understand your suggestion. I agree Poisson regression is used for count outcome variable. However, Poisson regression analysis model with robust variance estimate is used for binary outcome. We had tried to fit models like logistic regression, Binomial and Poisson regression analysis model with robust variance estimate. Even-though the three model can be used for binary outcome they have their own limitation. First we had fitted with logistic regression, then Binomial Model and finally Poisson regression analysis model with robust variance estimate. Poisson regression analysis model with robust variance estimate had gave n us a narrow Confidence interval and we choose this one. Since Confidence interval of logistic regression was larger we had selected Poisson regression analysis model with robust variance estimate. Regarding the variable we had checked the model fitness with AIC and BIC. There are many variables fitted to multivariate model, but during model fitness selection with stepwise a few variables are removed from the model and we checked the two model. Finally we left with few variables used in multivariate analyse.

Here is the link you can access for the detail information

1. Comparison of robustness to outliers between robust poisson models and log-binomial models when estimating relative risks for common binary outcomes: a simulation study **<https://link.springer.com/article/10.1186/1471-2288-14-82>**
2. **A Modified Poisson Regression Approach to Prospective Studies with Binary Data**<https://academic.oup.com/aje/article-abstract/159/7/702/71883>

Reviewer #2: Dietary Practices and Associated Factors among Pregnant Women in Haramaya District, Eastern Ethiopia (PONE-D-21-17077).
PLOS ONE Comments to the Author
1. Is the manuscript technically sound, and do the data support the conclusions?
The manuscript must describe a technically sound piece of scientific research with data that supports the conclusions. Experiments must have been conducted rigorously, with appropriate controls, replication, and sample sizes. The conclusions must be drawn appropriately based on the data presented.
Yes.
2. Has the statistical analysis been performed appropriately and rigorously?
Yes
3. Have the authors made all data underlying the findings in their manuscript fully available?
Yes
4. Is the manuscript presented in an intelligible fashion and written in Standard English?
No
5.  Reviewer #2 comments to the Author

Thank you a lot for your substantial effort. Really this comment is helpful to enrich the manuscript. Authors get your concern and critically looked your comment.

Considering the reviewer #3 has argued that“Meta-analysis was conducted on the dietary practices ” and suggested us to modify the research title to generate new evidence for scientific community and nutrition experts, we have tied to modify the research tittle to consumption of animal source food and associated factors among pregnant women in Eastern Ethiopia: A community-based study.

Additionally, this research is one objective of PhD dissertation work which was entitled “Prenatal Iron Status and Infants’ Birth Weight: Determinants of dietary practices, risk factors of iron deficiency, and infants’ birth weight and its association with maternal iron status in Haramaya district, eastern Ethiopia” by Meseret Belete Fite under supervention of the co-authored professors.

In this revised manuscript we have modified the title to “Consumption of animal source food and associated factors among pregnant women in Eastern Ethiopia: A community-based study.

For your information these are specific objectives the dissertation:

Specific objectives

4. To assess consumption of animal source food and associated factors among pregnant women in Eastern Ethiopia: A community-based study (Paper I)
5. To determine iron status and risk factors of iron deficiency among pregnant women in Haramaya district, eastern Ethiopia, 2021 (paper II)
6. Examine the associations between infants’ birth weight and maternal iron status in Haramaya district, eastern Ethiopia, 2021 (paper III)

Thus for this revised manuscript, we have two research question for our research . 1 what is the level of consumption of animal source food in haramya disrict. 2 what are the possible factors associated with consumption of animal source food. Actually all the detail of data for dietary practices of the pregnant women was collected as baseline for the cohort. However, as the the most common source of iron is animal source foods and we are interested to link the finding with the second objective(iron status and risk factors of iron deficiency) , we had to modified the the title .

Reviewer comments to the Author
Summary
The manuscript is good. However, it does need an edition by a native English speaker or Journal English language services. Because PLOS ONE does not copyedit accepted manuscripts; the authors should employ an editor to assist with ambiguous and grammatical errors that appear throughout the text. There are multiple grammar and sentence structure corrections that are required prior to publication.

**Authors’ responses:** Thank you very much. We appreciated your critical and thorough review of our manuscript and constructive comments that will improve our paper to its best. We have tried to correct typos and grammatical errors in the revised manuscript using language expert.

In title page And those authors listed above are also contributed equally to this work. Better if you use the symbol for the word and.

**Authors’ responses:**Thank you very much. We have tried to remove the statement “And Those authors listed above are also contributed equally to this work”in the revised manuscript and described the contribution of authors under the Authors’ contributions

Please in include line number. Table 2: Factors associated with dietary practice among pregnant women in Haramaya district, Eastern Ethiopia, 2021. Table title should be written at the start.

**Authors’ responses:** Thank you a lot for your substantial effort. We have tried to include tables (Table 2. Consumption frequency of ASF by pregnant women in Haramaya District, eastern Ethiopia, 2021 (n=448) and (Table 3:Factors associated with animal source food consumption among pregnant women in Eastern Ethiopia, 2021) in the revised manuscript

Abstract
In your abstract part, please change the word Introduction by Background. in general, you have to follow the author guideline of the journal.

**Authors’ responses:** Thank you a lot for your substantial effort. We have tried to review PloseOne guideline and it uses introduction. I have also published papers on this journal using the word Introduction. If I am still not correct, I will consider it.

In Method part Sectional study design what does it mean? Conclusion, in that dietary practice of pregnant women is sub-optimal. What is your reference to say sub-optimal.

**Authors’ responses :** Thank you very much for your substantial comments. We have corrected to the study design to “A community-based cross-sectional study was conducted “ I the revised manuscript
Introduction
The introduction part is unclear. It should be written again coherently.

**Authors’ responses:** Thank you so much. We appreciated your valuable comments. We have tried to modify the introduction part in the revised manuscript.

Method
Data collection and Measurement part is vague. Your operational definition is not clear. It should be rewritten again. Additionally, High DDS: Tercile calculated from food groups, and the highest tercile was considered as a high DDS, whereas the rest two lower terciles were taken as a low DDS.
High DDS: terciles were calculated from food groups, and the highest tercile was considered as a high DDS, whereas the rest two lower terciles were taken as low DDS

**Authors’ responses:** Thank you so much. We appreciated your comments. We have tried to rewrite data collection and measurement in the revised manuscript

(25) . Please remove redundancy.
     Factors Associated with Dietary Practice in this part you have written only the word table. But, you should include table number.
     Again, Table 2: Factors associated with dietary practice among pregnant women in Haramaya district, Eastern Ethiopia,2021 is written at the end of your table which is not acceptable.
     Table 2 in footnote you have incorporated definitions like Appropriate dietary practice: is defined as the consumption of at least four meals daily, high DDS, high FVS and high ASF; otherwise, Inappropriate dietary practice and Elementary school: Grade 1-8. But, it is redundant and better if you write it in your measurement part only.

**Authors’ responses:** Thank you so much. We appreciated your valuable comments. We have tied to define the measurement of consumption of foods from an animal source as outcome variable in the revised manuscript. We have stated as “ The consumption of foods from an animal source (ASF) was estimated by counting the frequency of each food from animal sources that pregnant women ate over a reference period. Animal source foods score was also converted into tertile and the highest tertile used to label as "highs, while the two lower tertiles combined were defined as "low" ASF”

(26) Results
     Table 1. Educational level of women is not standard classification.

**Authors’ responses:** Thank you so much. We appreciated your valuable comments. We have tried re-classified educational level of women in standard classification in the revised manuscript as (Educational level of the woman 1= Can’t read or write, 2=Read or write, 3=Formal education)

Additionally, in your footnote of your table you have included definitions like Housewife: Women whose activities are in home and not participated in yielding family financial income
Formal education+: Refers grade 1 and above
Wealth index quintile: Was computed from wealth score of the households and used to label households wealth status to five categories
Family Size: Refers to total number of family members living together
Parity: Refers to the number of births the mother experienced after 28 weeks whatever the status of the newborn is (WHO,2015). But, if you want to incorporate these definitions it is better to include in the measurement part. Finally, what is 1 mean?

**Authors’ responses:** Thank you so much. We appreciated your valuable comments. We have removed the definition from the footnote of the table in the revised manuscript.

Discussion
Adequate nutrition in pregnancy is important for maternal and child health (3). There is mounting evidences that, insufficient consumption of balanced and quality diet during pregnancy has a great effect on fetus health and development and may also result to poor birth outcome (4). At beginning of pregnancy many women lack sufficient micronutrients stores to meet physiological requirement (1) and they are more vulnerable to malnutrition (29). Several epidemiological studies indicated inappropriate dietary practice is significant contributor to maternal under nutrition and micronutrients deficiency in resource limited countries (30) .Your discussion part is very broad and somewhat not coherent. It needs a major revision.

**Authors’ responses:** Thank you a lot for your substantial concern. We have made a major revision of the discussion in the revised manuscript

Conclusion
Your conclusion is somewhat vague. Better if you write it clearly based on your finding.

**Authors’ responses:** Thank you a lot for your substantial concern. We have made a major revision of the conclusion in the revised manuscript

References
Your reference is not written according to PLoS one in addition to some of them is very old. Please, review your references and adjust agreeing the PLOS specifications. For instance, you write some references in their complete journal’s title, whereas others were abbreviated.
6. PLOS authors have the option to publish the peer review history of their article (what does this mean?). If published, this will include your full peer review and any attached files.
If you choose “no”, your identity will remain anonymous but your review may still be made public.
Do you want your identity to be public for this peer review?
No

Reviewer #3: Dear Dr Meseret Belete
I am glad enough on your area of interest to do research on “Dietary Practices and Associated Factors among Pregnant Women in Haramaya District, Eastern Ethiopia” a means of prevention of feto-maternal adverse outcomes. Unfortunately, the whole of the manuscript lacks novelty and needs extensively edition due to a lot of typographical and grammatical errors which should be corrected. Otherwise unable proceed to further review. Again revise the word and line spacing across the manuscript. E.g., “asses” should be written as “assess”, “ meal frequency are measures usually” to be revised as “meal frequency measures are usually”, and lots punctuation errors.

**Authors’ responses:** Thank you very much. We appreciated your critical and thorough review of our manuscript and constructive comments that will improve our paper to its best. We have tried to correct typos and grammatical errors in the revised manuscript using language expert.

Why you are intended to do this research due to many studies including meta-analysis on the area and what new evidence you provide for scientific community and nutrition experts? [https://pubmed.ncbi.nlm.nih.gov/32855822](https://pubmed.ncbi.nlm.nih.gov/32855822) and [https://pubmed.ncbi.nlm.nih.gov/34111140](https://pubmed.ncbi.nlm.nih.gov/34111140).

Thank you a lot for your substantial effort. Really this comment is helpful to enrich the manuscript. Authors get your concern and critically looked your comment.

Considering the reviewer #3 has argued that“Meta-analysis was conducted on the dietary practices ” and suggested us to modify the research title to generate new evidence for scientific community and nutrition experts, we have tied to modify the research tittle to consumption of animal source food and associated factors among pregnant women in Eastern Ethiopia: A community-based study.

Additionally, this research is one objective of PhD dissertation work which was entitled “Prenatal Iron Status and Infants’ Birth Weight: Determinants of dietary practices, risk factors of iron deficiency, and infants’ birth weight and its association with maternal iron status in Haramaya district, eastern Ethiopia” by Meseret Belete Fite under supervention of the co-authored professors.

In this revised manuscript we have modified the title to “Consumption of animal source food and associated factors among pregnant women in Eastern Ethiopia: A community-based study.

For your information these are specific objectives the dissertation:

Specific objectives

7. To assess consumption of animal source food and associated factors among pregnant women in Eastern Ethiopia: A community-based study (Paper I)
8. To determine iron status and risk factors of iron deficiency among pregnant women in Haramaya district, eastern Ethiopia, 2021 (paper II)
9. Examine the associations between infants’ birth weight and maternal iron status in Haramaya district, eastern Ethiopia, 2021 (paper III)

Thus for this revised manuscript, we have two research question for our research . 1 what is the level of consumption of animal source food in haramya disrict. 2 what are the possible factors associated with consumption of animal source food. Actually all the detail of data for dietary practices of the pregnant women was collected as baseline for the cohort. However, as the the most common source of iron is animal source foods and we are interested to link the finding with the second objective(iron status and risk factors of iron deficiency) , we had to modified the the title .
Chat chewing and restricted food intake is known factor for decrease diatery practice and might be having collinearly effect. How you control the effect of chat chewing on food restriction and dietary practice?

**Authors’ responses:** Thank you a lot for your substantial concern. We have tried to carry out multico-linearity test to see the correlation between independent variables by using the standard error and collinearity statistics (variance inflation factors >10 and standard error >2 were considered as suggestive of the existence of multi co-linearity). During analysis we found the “restricted food intake” had collinearly effect with Chat chewing. Thus we removed restricted food intake from the model and included chat chewing in the final model in revised manuscript.

If you have the data better to do analysis by fitting other new variables in your regression. Hence, the variables you got are already explored by other studies. It was better to do mixed studies to explore qualitative barriers of adherence to recommendations of dietary diversity of pregnant women. Again, it needs project to alleviate the problem than repeated single research.

**Authors’ responses:** Thank you a lot for your substantial concern. Since we have not collected qualitative information , we have tied to recommend the need of future further qualitative study using qualitative to explore barriers in the revised manuscript.

You suggest in the background “environmental and psychological factors in pregnancy are not well-known in this setup empirically”, but we can’t access it in the analysis.

**Authors’ responses:** Thank you a lot for your substantial concern. We have tried to remove the description of statement “environmental and psychological factor” in the revised manuscript.

How to measure dietary diversity among pregnant women, if you included those who were restricted of dietary/ food intake? Hence, you already put as a factor.
Please describe the possible determinants of dietary practice in Ethiopia and other settings?

**Authors’ responses:**Thank you so much. We appreciated your comments. We have tied to modify to the identify the determinants of animal source food consumption among pregnant women

Why intended to use Poisson regression analysis model?

Thank you so much. We appreciated your comments. We had fitted different analytical models and we have applied binary logistic regression model in revised manuscript. This model gave us narrow confidence interval , it was appropriate for our case.

However for your concern we wanted to give a brief why we used Poisson regression model in previous submission. We used in, Poisson regression analysis model with robust variance estimate for analysis previous submission . I understand your suggestion. I agree Poisson regression is used for count outcome variable. However, Poisson regression analysis model with robust variance estimate is used for binary outcome. We had tried to fit models like logistic regression, Binomial and Poisson regression analysis model with robust variance estimate. Even-though the three model can be used for binary outcome they have their own limitation. First we had fitted with logistic regression, then Binomial Model and finally Poisson regression analysis model with robust variance estimate. Poisson regression analysis model with robust variance estimate had gave n us a narrow Confidence interval and we choose this one. Since Confidence interval of logistic regression was larger we had selected Poisson regression analysis model with robust variance estimate. Regarding the variable we had checked the model fitness with AIC and BIC. There are many variables fitted to multivariate model, but during model fitness selection with stepwise a few variables are removed from the model and we checked the two model. Finally we left with few variables used in multivariate analyse.

Here is the link you can access for the detail information

1. Comparison of robustness to outliers between robust poisson models and log-binomial models when estimating relative risks for common binary outcomes: a simulation study **<https://link.springer.com/article/10.1186/1471-2288-14-82>**
2. **A Modified Poisson Regression Approach to Prospective Studies with Binary Data**<https://academic.oup.com/aje/article-abstract/159/7/702/71883>

Did you include women with emesis gravidarum before 12 weeks? And what specific illness was excluded in your actual data collection?

**Authors’ responses:** Thank you so much. We appreciated your comments. As we described in the Study settings section of the manuscript “The study was embedded into the Haramaya Health Demographic Surveillance and Health Research Centre (HDS-HRC), which was established in 2018. In HDS-HRC 2306 pregnant women were followed. We had included 19 confirmed first trimester (before 12 week) pregnant women who had been registered in HDS-HRC and on follow up. The HDS-HRC frequently collect and update the information of women who were under the project catchment.

We had excluded pregnant women who were critically ill during data collection in this study. However Since the study was the prospective cohort study,to control the confounding effect of the inflammation for the evaluation of iron status (Serum ferreitin), we had performed highly sensitive C-protein reactive (hsCRP) at Ethiopian public health institute(EPHI).Accordingly, highly sensitive C-protein reactive (hsCRP) was analyzed by Roche/Hitachi cobas 6000 (c501): (German, Japan Cobas 6000 series of Roche) fully automated clinical chemistry analyzer. The tests were performed by trained and experienced medical laboratory technologist.

What does that mean 15.18% of pregnant women have dietary practice in your abstract section? appropriate dietary practice or HDD?

**Authors’ responses:**Thank you so much. We appreciated your comments. We have tied to use frequency of animal source food consumption consistently in abstract section, result and discussion in the revised manuscript. We reported “The prevalence of the high frequency of animal source food consumption among the study participants was 24.78% (95% CI= 21%-29%)”

The write up of the results section needs extensive edition and less attractive the reader
Remove repeated words and used the journal guideline for the submission. E.g. put the table after the reference section with Table Legends, and double line spacing.
In discussion, you have expected to put your findings and compare with other studies and also suggest the possible implications of your study.

A**uthors’ responses:**Thank you so much. We appreciated your comment. We have made extensive edition the whole sections of the revised manuscript for best attractive for the readers by language experts.

6. PLOS authors have the option to publish the peer review history of their article ([what does this mean?](https://journals.plos.org/plosone/s/editorial-and-peer-review-process#loc-peer-review-history)). If published, this will include your full peer review and any attached files.


If you choose “no”, your identity will remain anonymous but your review may still be made public.


**Do you want your identity to be public for this peer review?** For information about this choice, including consent withdrawal, please see our [Privacy Policy](https://www.plos.org/privacy-policy).

Reviewer #1: **Yes:** DEREJE TSEGAYE

Reviewer #2: No

Reviewer #3: **Yes:** melaku desta

[NOTE: If reviewer comments were submitted as an attachment file, they will be attached to this email and accessible via the submission site. Please log into your account, locate the manuscript record, and check for the action link "View Attachments". If this link does not appear, there are no attachment files.]

While revising your submission, please upload your figure files to the Preflight Analysis and Conversion Engine (PACE) digital diagnostic tool, [https://pacev2.apexcovantage.com/](https://pacev2.apexcovantage.com/). PACE helps ensure that figures meet PLOS requirements. To use PACE, you must first register as a user. Registration is free. Then, login and navigate to the UPLOAD tab, where you will find detailed instructions on how to use the tool. If you encounter any issues or have any questions when using PACE, please email PLOS at [figures@plos.org](mailto:figures@plos.org). Please note that Supporting Information files do not need this step
