# Supplementary material for: Consumption of animal source food and associated factors among pregnant women in eastern Ethiopia: A community-based study
Source: PLoS One. 2022 Jun 17;17(6):e0270250. doi: 10.1371/journal.pone.0270250 (PMC9205500; doi:10.1371/journal.pone.0270250)
Supplement: Attachment — Submitted filename: Response to Reviewers revised.docx [file pone.0270250.s003.docx]

AUTHORS’ RESPONSES TO THE EDITORS AND REVIEWERS’ COMMENTS AND SUGGESTIONS

| PLOS ONE <em@editorialmanager.com> | Dec 14, 2021, 9:45 AM |  |    |
| --- | --- | --- | --- |
| to me  | | |  |

PONE-D-21-17077

PONE-D-21-17077R1

Consumption of animal source food and associated factors among pregnant women in Eastern Ethiopia: A community-based study

Dear Melaku Desta !

Guest Editor
PLOS ONE

Editor comments and authors’ response

Dear authors of manuscript
It is highly worthful paper and an extensive revision was made based on the reviewers comment. Despite, to accept it for publication still needs further revision mainly on the typographical errors across the manuscript. Thus, lets correct the grammar errors.
Again, you have put the problem statement in clearly manner in the context of LMIC and Ethiopia instead of comparing with USA.

**Authors’ responses to** Editor Comments

**Authors’ responses:** Thank you a lot for your substantial effort. Really this comment is helpful to enrich the manuscript. First of all we would like to acknowledge you for your constructive and valuable comments and helpful suggestions that helped us to improve and enrich our manuscript. On behalf of my co-authors, I thank you for your consideration of this re-submission. We have tried to correct typos and grammatical errors in the revised manuscript using language expert.

Reviewer comments

Reviewer #1: The authors have tried to address the comments. However, the manuscript has a lot of grammatical errors. Needs proof reading.

**Authors’ responses to** reviewer 1 comments

**:** Thank you very much. We appreciated your critical and thorough review of our manuscript and constructive comments that will improve our paper to its best. We have tried to correct typos and grammatical errors in the revised manuscript using language expert
