# Supplementary material for: Consumption of animal source food and associated factors among pregnant women in eastern Ethiopia: A community-based study
Source: PLoS One. 2022 Jun 17;17(6):e0270250. doi: 10.1371/journal.pone.0270250 (PMC9205500; doi:10.1371/journal.pone.0270250)
Supplement: Attachment — Submitted filename: Response to Reviewers revised 3.docx [file pone.0270250.s004.docx]

AUTHORS’ RESPONSES TO THE EDITORS AND REVIEWERS’ COMMENTS AND SUGGESTIONS

PONE-D-21-17077R1

Consumption of animal source food and associated factors among pregnant women in Eastern Ethiopia: A community-based study

Dear Melaku Desta !

Guest Editor
PLOS ONE

Editor comments and authors’ response

Thank you for submitting your manuscript to PLOS ONE. After careful consideration, we feel that it has merit but does not fully meet PLOS ONE’s publication criteria as it currently stands. Therefore, we invite you to submit a revised version of the manuscript that addresses the points raised during the review process.

Please submit your revised manuscript by Jul 04 2022 11:59PM. If you will need more time than this to complete your revisions, please reply to this message or contact the journal office at [plosone@plos.org](mailto:plosone@plos.org). When you're ready to submit your revision, log on to <https://www.editorialmanager.com/pone/> and select the 'Submissions Needing Revision' folder to locate your manuscript file.

Please include the following items when submitting your revised manuscript:

- A rebuttal letter that responds to each point raised by the academic editor and reviewer(s). You should upload this letter as a separate file labeled 'Response to Reviewers'.
- A marked-up copy of your manuscript that highlights changes made to the original version. You should upload this as a separate file labeled 'Revised Manuscript with Track Changes'.
- An unmarked version of your revised paper without tracked changes. You should upload this as a separate file labeled 'Manuscript'.

If you would like to make changes to your financial disclosure, please include your updated statement in your cover letter. Guidelines for resubmitting your figure files are available below the reviewer comments at the end of this letter.

If applicable, we recommend that you deposit your laboratory protocols in [protocols.io](http://protocols.io/) to enhance the reproducibility of your results. Protocols.io assigns your protocol its own identifier (DOI) so that it can be cited independently in the future. For instructions see: <https://journals.plos.org/plosone/s/submission-guidelines#loc-laboratory-protocols>. Additionally, PLOS ONE offers an option for publishing peer-reviewed Lab Protocol articles, which describe protocols hosted on [protocols.io](http://protocols.io/). Read more information on sharing protocols at <https://plos.org/protocols?utm_medium=editorial-email&utm_source=authorletters&utm_campaign=protocols>.

We look forward to receiving your revised manuscript.

Kind regards,

Melaku Desta
Guest Editor
PLOS ONE

Journal Requirements:

Please review your reference list to ensure that it is complete and correct. If you have cited papers that have been retracted, please include the rationale for doing so in the manuscript text, or remove these references and replace them with relevant current references. Any changes to the reference list should be mentioned in the rebuttal letter that accompanies your revised manuscript. If you need to cite a retracted article, indicate the article’s retracted status in the References list and also include a citation and full reference for the retraction notice.

Additional Editor Comments (if provided):

Dear authors of manuscript
It is highly worthful paper and an extensive revision was made based on the reviewers comment. Despite, to accept it for publication still needs further revision mainly on the typographical errors across the manuscript. Thus, lets correct the grammar errors.
Again, you have put the problem statement in clearly manner in the context of LMIC and Ethiopia instead of comparing with USA.

**Authors’ responses to** Editor Comments

**Authors’ responses:** Thank you a lot for your substantial effort. Really this comment is helpful to enrich the manuscript. First of all we would like to acknowledge you for your constructive and valuable comments and helpful suggestions that helped us to improve and enrich our manuscript. On behalf of my co-authors, I thank you for your consideration of this re-submission. We have tried to correct typos and grammatical errors in the revised manuscript using language expert. We have again tried to revise the problem statement in in revised manuscript with the context of LMIC. We have tried to highlight the change in on track change manuscript. We have stated clearly in introduction part paragraph two line 3-11. Here below are the updated statements.

“Inadequate dietary consumption in pregnancy is an important contributor to global maternal malnutrition in less developed countries (19). A previous study showed that pregnant women in developing countries suffer from energy deficiencies due to comparatively inadequate energy intake (20). Cultural norms and customs govern dietary intake behaviors in several traditional societies comprising critical life stages such as pregnancy (21). Meat and egg are taboo among pregnant women in South Eastern Nigeria (22). Ethiopia is noted to have one of the substantial livestock populations globally (23). Nevertheless, a low intake of meat, fish, fruits, and some vegetables during pregnancy is reported in (24).

[Note: HTML markup is below. Please do not edit.]

Reviewers' comments:

Reviewer's Responses to Questions

**Comments to the Author**

1. If the authors have adequately addressed your comments raised in a previous round of review and you feel that this manuscript is now acceptable for publication, you may indicate that here to bypass the “Comments to the Author” section, enter your conflict of interest statement in the “Confidential to Editor” section, and submit your "Accept" recommendation.

Reviewer #1: (No Response)

Reviewer #2: All comments have been addressed

2. Is the manuscript technically sound, and do the data support the conclusions?

The manuscript must describe a technically sound piece of scientific research with data that supports the conclusions. Experiments must have been conducted rigorously, with appropriate controls, replication, and sample sizes. The conclusions must be drawn appropriately based on the data presented.

Reviewer #1: Yes

Reviewer #2: Yes

3. Has the statistical analysis been performed appropriately and rigorously?

Reviewer #1: Yes

Reviewer #2: Yes

4. Have the authors made all data underlying the findings in their manuscript fully available?

The [PLOS Data policy](http://www.plosone.org/static/policies.action#sharing) requires authors to make all data underlying the findings described in their manuscript fully available without restriction, with rare exception (please refer to the Data Availability Statement in the manuscript PDF file). The data should be provided as part of the manuscript or its supporting information, or deposited to a public repository. For example, in addition to summary statistics, the data points behind means, medians and variance measures should be available. If there are restrictions on publicly sharing data—e.g. participant privacy or use of data from a third party—those must be specified.

Reviewer #1: Yes

Reviewer #2: Yes

5. Is the manuscript presented in an intelligible fashion and written in standard English?

PLOS ONE does not copyedit accepted manuscripts, so the language in submitted articles must be clear, correct, and unambiguous. Any typographical or grammatical errors should be corrected at revision, so please note any specific errors here.

Reviewer #1: No

Reviewer #2: Yes

6. Review Comments to the Author

Please use the space provided to explain your answers to the questions above. You may also include additional comments for the author, including concerns about dual publication, research ethics, or publication ethics. (Please upload your review as an attachment if it exceeds 20,000 characters)

Reviewer #1: Th authors have tried to address the comments. However, the manuscript has a lot of grammatical errors. Needs proof reading.

**Authors’ responses to** reviewer 1 comments

Thank you very much. We appreciated your critical and thorough review of our manuscript and constructive comments that will improve our paper to its best. We have tried to correct typos and grammatical errors in the revised manuscript using language expert. We have tried to highlight the change in on track change manuscript.

Reviewer #2: Authors have made a significant improvement in their revised document. It now looks amended and clear to the reader.

**Authors’ responses to** reviewer 2 comments

Thank you very much for your appreciation. We have also tried to address the points raised by editor and reviewer 1 comments in the revised manuscript.
